# First report of *Toxoplasma gondii* seroprevalence in wild-caught Caribbean African green monkeys

**DOI:** 10.1186/s13071-014-0571-x

**Published:** 2014-12-10

**Authors:** Clare M Hamilton, Frank Katzer, Amy Beierschmitt, Esteban Soto, Elisabeth A Innes, Patrick J Kelly

**Affiliations:** 1 Moredun Research Institute, Pentlands Science Park, Bush Loan, Edinburgh, EH26 0PZ UK; 2 Ross University School of Veterinary Medicine, PO Box 334, Basseterre, St. Kitts Saint Kitts and Nevis; 3 Behavioural Science Foundation, PO Box 428, Estridge Estate, Basseterre, St. Kitts Saint Kitts and Nevis

**Keywords:** *Toxoplasma gondii*, Vervet monkey, *Chlorocebus sabaeus*, Seroprevalence, St. Kitts

## Abstract

**Background:**

*Toxoplasma gondii* is a protozoan parasite capable of infecting all warm-blooded animals. Humans can become infected by ingesting infective oocysts from the environment or contaminated food or water, or by ingesting tissue cysts in undercooked infected meat or by handling infected meat. Caribbean African green monkeys (*Chlorocebus sabaeus*) are present in large numbers on the island of St. Kitts in the Caribbean, and it is not uncommon for these animals to be trapped and eaten by islanders. The aim of this study was to determine *T. gondii* infection in Caribbean African green monkeys.

**Findings:**

Sera collected from 79 wild-caught Caribbean African green monkeys were examined for *T. gondii* antibodies by ELISA. Antibodies were detected in 38 out of 79 (48.1%) monkeys. Significantly more females were infected than males but there was no significant effect of age or location on antibody status.

**Conclusions:**

Results indicate that Caribbean African green monkeys can be infected with *T. gondii* and that there is widespread environmental contamination of St. Kitts with oocysts. These monkeys could present a potential source of *T. gondii* infection if their meat is consumed undercooked. This is the first report of *T. gondii* antibodies in this species.

## Findings

### Background

*Toxoplasma gondii* is a ubiquitous protozoan parasite capable of infecting all warm-blooded animals [1]. In intermediate hosts, such as humans and non-human primates, the parasite develops into a cystic form in the tissues which may persist in a viable state for the lifetime of the host. Humans become infected with *T. gondii* by ingesting tissue cysts from raw or undercooked meat, or by ingesting oocysts (shed in infected cat faeces) from contaminated food or water or directly from the environment.

Toxoplasmosis has a wide spectrum of clinical responses following infection which ranges from acute fatal disease, congenital disease, behavioural changes and no obvious clinical signs [2]. The outcome of *T. gondii* infection may be influenced by factors such as the definitive host, whether or not the host species has evolved alongside the cat, how the immune system responds to the infection, and the influence of the parasite strain. Most *T. gondii* infections are mild or asymptomatic but the differing pathogenicity of the parasite is evident in lemurs [3], Australian marsupials [4] and new world monkeys [5] where the parasite is highly virulent and can cause severe clinical symptoms and even death in a primary infection. Unlike new world monkeys, old world monkeys, such as the African green monkeys (*Chlorocebus sabaeus*), are less susceptible to clinical disease and there are few reports of toxoplasmosis in these hosts.

St. Kitts is a small island located in the Eastern Caribbean with a population of approximately 35,000 people. African green monkeys (AGM) were introduced to the island in the 1700s when they were transported across the Atlantic from West Africa during the slave trade. Many slavers brought them as pets or to be sold or traded for goods [6]. Today, there are populations of Caribbean AGM on St. Kitts, Nevis and Barbados. On St. Kitts, the population of Caribbean AGM is estimated at 55,000 which significantly outnumbers the human population. Their large numbers can be problematic, with habitat and crop destruction impacting on the livelihoods of local residents. They are considered a pest and it is not uncommon for the monkeys to be trapped and killed, and their meat consumed as bush meat [7].

Previous work on St. Kitts demonstrated a high *T. gondii* seroprevalence in cats [8,9] and small ruminants [10], suggesting widespread environmental contamination with oocysts. Although we could find no report of *T. gondii* infection in AGM, these animals occur throughout St. Kitts and may be sentinels for environmental contamination. To provide further data on the distribution of *T. gondii* on St. Kitts, we examined sera from wild monkeys trapped around the island and report our results below.

### Methods

**Figure 1:**
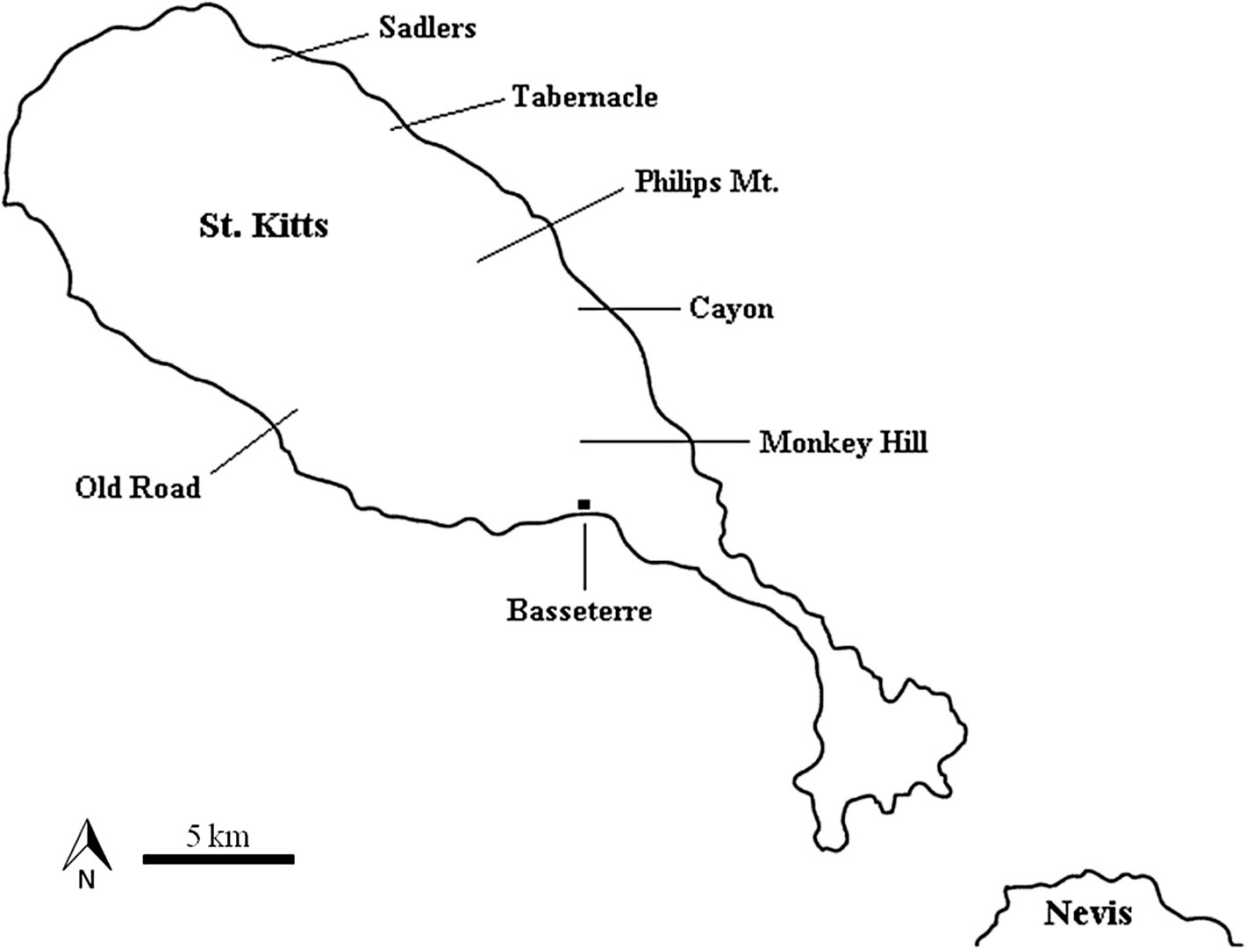
**Map of St. Kitts depicting the trapping sites of the wild-caught monkeys and the island capital, Basseterre.**

Seventy nine Caribbean AGM (*Chlorocebus sabaeus*) were trapped at 7 different locations throughout St. Kitts (Figure 1) and brought to a quarantine site which directly serves the Behavioural Science Foundation, St. Kitts [11]. Animals were anesthetized and 2 ml of whole blood was collected from the femoral vein as part of a larger study which received ethical approval from the Institutional Animal Care and Use Committee (IACUC), Ross University School of Veterinary Medicine, St. Kitts [11]. Further ethical approval for screening the sera for *T. gondii* antibodies was granted by IACUC. Sera were separated and stored at -80°C.

All sera were examined for *T. gondii* antibodies using an in-house ELISA which has been reported to have a sensitivity of 99% and a specificity of 99.4% [12], with modifications. In brief, 96-well microtitre plates were coated overnight with 6 μg/ml solubilised RH antigen [13], washed with PBST (PBS with 0.05% Tween-20) and incubated for 1 hr at room temperature (approximately 25°C) after addition of 100 μl test or control sera (diluted 1:100 in 1% BSA in PBST) per well. Following washing, 100 μl HRP-conjugated Protein G (Life Technologies Ltd, UK), diluted 1:10,000 in PBST with 1% BSA, was added to each well and plates incubated for 1 h at room temperature. ELISAs were developed with TMB and reactions stopped with 2 M H_2_SO_4_ before ODs were read at 450 nm. Control sera were pooled samples of 5 positive and 5 negative human serum samples from a previous study (Burrells et al. unpublished observations). For each plate, the cut-off value was calculated as two times the percent positivity of the negative control serum relative to the positive control serum (i.e. 2 × (average negative control sera OD/average positive control sera OD) × 100) [14].

Effects of gender, age and location on *T. gondii* antibody status were investigated using a general linear model. A *P* value of < 0.05 was deemed significant.

### Results

**Figure 2:**
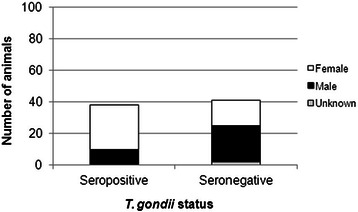
**Number of** ***T. gondii*** **seropositive and seronegative monkeys.**

Antibodies to *T. gondii* were detected in 38 out of 79 (48.1%; 95% confidence interval (CI): 37.4-59.0%) Caribbean African green monkeys (Figure 2). Of the 77 monkeys which had their gender recorded, 33 were male and 44 were female. Of the 38 *Toxoplasma*-positive monkeys, 28 (73.7%; CI: 58.0-85.0%) were female and 10 (26.3%; CI: 15.0-42.0%) were male (Figure 2). Females were significantly more likely to be seropositive than males (*P* = 0.04). Monkeys were caught from 7 locations across the island, and each site had at least one positive monkey (Table 1). There was no significant affect of age or location on *T. gondii* antibody status.

**Table 1:** **Geographical distribution of** ***T. gondii*** **-positive monkeys in St. Kitts**

**Island location**	**n tested**	**n positive**	**% positive (CI)**
Cayon	8	4	50.0 (21.5-78.5%)
Monkey Hill	3	1	33.3 (6.2-79.2%)
Old Road	30	14	46.7 (30.2-63.9%)
Phillips Mt.	9	4	44.4 (18.9-73.3%)
Saddlers Mt.	19	9	47.4 (27.3-68.3%)
Saddlers	3	3	100.0 (43.9-100.0%)
Tabernacle	6	2	33.3 (9.7-70.0%)
Unknown	1	1	100.0 (20.7-100.0%)
TOTAL	79	38	

### Discussion

This is the first study to show evidence of *T. gondii* infections in green monkeys. Green monkeys are opportunistic omnivores but in St. Kitts a large component of their diet consists of wild fruits, flowers and leaves. The high seroprevalence reported in the present study suggests a high level of environmental contamination with oocysts shed in infected cat faeces. Previous studies on St. Kitts have demonstrated a very high seroprevalence of *T. gondii* in both domestic and feral cat populations [8,9], suggesting that there could be wide spread contamination with oocysts. Following a primary infection, cats may shed up to 55 million oocysts per day into the environment [15]. In the warm temperatures of St. Kitts, these oocysts may sporulate and become infective in less than 1 day if there is also sufficient humidity and aeration [16]. Sporulated oocysts of *T. gondii* are very resistant to environmental conditions and can remain infective in moist soil or sand for up to 18 months [17]. Although previous studies on cats in St. Kitts focused on one area of the island (Basseterre), local experience shows feral and free-roaming cats are present all over the island and may contribute to dissemination of *T. gondii* oocysts in the environment thus presenting a potential route of transmission to the monkeys. This was reflected in the finding that at least one monkey from each island location was seropositive for *T. gondii*.

Green monkeys also consume insects and earthworms in their diet, which could increase their chance of ingesting any oocysts that may be present in the soil. It is possible that the monkeys may also have been infected congenitally, as this route of transmission has been reported in non-human primates. Experimental infection of rhesus macaques (*Macaca mulatta*) with *T. gondii* resulted in abortion as well as congenital and ocular toxoplasmosis [18,19]. The youngest monkey in the present study was 3 years old, so it is not possible to surmise whether congenital infection is a significant transmission route in these animals.

In this study, although the sample size was small, seroprevalence was significantly higher in female monkeys than in male monkeys. The average estimated age of the female monkeys was 5.7 years, and 6.8 years for the male monkeys, so it is unlikely that age played a role in the differing seroprevalence rates. Indeed, there was no significant effect of age on antibody status of the monkeys. Higher *T. gondii* seroprevalence in females has been reported for other animals, including donkeys [20] and pigs [21]; however, gender is not thought to be a significant risk factor for *T. gondii* infection.

It is well documented that new world monkeys, such as the squirrel monkey (*Saimiri sciureus*), are more susceptible to toxoplasmosis than old world monkeys and can suffer fatal multisystemic disease [5,22]. There are few reports of *T. gondii* in old world monkeys [23-25] and none reports clinical signs of toxoplasmosis. To our knowledge, our study is the first to indicate that Caribbean AGM can be infected with *T. gondii*.

With an estimated population of 55,000, the Caribbean AGM on St. Kitts are a significant pest to farmers and islanders growing their own fruit and vegetables [7]. Consequently, the monkeys are not uncommonly killed and sometimes eaten as bush meat [7]. Our finding of seropositive monkeys suggests they could be a source of *T. gondii* if they harbour viable tissue cysts and their meat is consumed raw or undercooked. Consumption of undercooked infected meat has been identified as a significant risk factor in several case-control studies on *Toxoplasma* infection in humans [26]. To prevent foodborne transmission, meat should be thoroughly cooked (67°C or higher) before consumption [27] and it should not be tasted during preparation or cooking [26].

### Conclusions

In conclusion, this is the first report of *T. gondii* antibodies in Caribbean AGM and the high exposure rates we found on St. Kitts indicate widespread environmental contamination of the island with oocysts. Further studies are in planning to determine the role bush meat might play in the epidemiology of the organism on St. Kitts.
